# Safety and Antihypertensive Effect of Selara® (Eplerenone): Results from a Postmarketing Surveillance in Japan

**DOI:** 10.1155/2016/5091951

**Published:** 2016-10-24

**Authors:** Shoko Takahashi, Megumi Hiramatsu, Shinichi Hotta, Yukie Watanabe, Osamu Suga, Yutaka Endo, Isamu Miyamori

**Affiliations:** ^1^Medical Affairs, Global Established Pharma Business, Pfizer Japan Inc., Shinjuku Bunka Quint Building, 3-22-7 Yoyogi, Shibuya-ku, Tokyo 151-8589, Japan; ^2^PMS Planning & Operation, Pfizer Japan Inc., Shinjuku Bunka Quint Building, 3-22-7 Yoyogi, Shibuya-ku, Tokyo 151-8589, Japan; ^3^Clinical Informatics and Innovation, Pfizer Japan Inc., Shinjuku Bunka Quint Building, 3-22-7 Yoyogi, Shibuya-ku, Tokyo 151-8589, Japan; ^4^Medical Writing and Document Management, Pfizer Japan Inc., Shinjuku Bunka Quint Building, 3-22-7 Yoyogi, Shibuya-ku, Tokyo 151-8589, Japan; ^5^Clinical Statistics, Pfizer Japan Inc., Shinjuku Bunka Quint Building, 3-22-7 Yoyogi, Shibuya-ku, Tokyo 151-8589, Japan; ^6^University of Fukui, 23-3 Matsuokashimoaizuki, Eiheiji-cho, Yoshida-gun, Fukui Prefecture 910-1193, Japan

## Abstract

Prospective postmarketing surveillance of Selara (eplerenone), a selective mineralocorticoid receptor antagonist, was performed to confirm its safety and efficacy for hypertension treatment in Japan. The change in blood pressure after initiation of eplerenone treatment was also examined. Patients with essential hypertension who were eplerenone-naïve were recruited regardless of the use of other antihypertensive drugs. For examination of changes in blood pressure, patients were excluded if eplerenone was contraindicated or used off-label. Patients received 50–100 mg of eplerenone once daily and were observed for 12 weeks. No treatments including antihypertensive drugs were restricted during the surveillance period. Across Japan, 3,166 patients were included for safety analysis. The incidence of adverse drug reactions was 2.4%. The major adverse drug reactions observed were hyperkalemia (0.6%), dizziness, renal impairment, and increased serum potassium (0.2% each). The mean systolic blood pressure decreased from 152.1 ± 19.0 mmHg to 134.8 ± 15.2 mmHg at week 12, and the mean diastolic blood pressure decreased from 85.8 ± 13.7 mmHg to 77.7 ± 11.4 mmHg. There were no significant new findings regarding the type or incidence of adverse reactions, and eplerenone had a clinically significant antihypertensive effect, leading to favorable blood pressure control.

## 1. Introduction

Hypertension is a major public health issue in many countries. In Japan, 53% of individuals aged 40–74 years and 79% of those aged 75 years and older were diagnosed with hypertension based on systolic blood pressure (SBP) ≥ 140 mmHg, diastolic blood pressure (DBP) ≥ 90 mmHg, and/or treatment with an antihypertensive drug, according to the 2013 National Health and Nutrition Survey published by the Ministry of Health, Labor, and Welfare [1]. The risk for cardiovascular events has been shown to be high among patients with hypertensive conditions, particularly among those who have comorbidities such as diabetes, chronic kidney disease (CKD), metabolic syndrome, cerebrovascular disorders, or organ dysfunction including heart disease. Therefore, it is important to provide guidance for lifestyle modifications and to administer strict treatment with antihypertensive drugs according to the target blood pressure (BP) levels, depending on the comorbidities [2]. The Eighth Joint National Committee [3], American Heart Association/American College of Cardiology/Centers for Disease Control and Prevention [4], and 2014 Guidelines for the Management of Hypertension by the Japanese Society of Hypertension (JSH 2014) [2] have recommended diuretics, calcium channel blockers (CCBs), angiotensin II receptor blockers (ARBs), and angiotensin-converting enzyme (ACE) inhibitors as first-line antihypertensive drugs [5]. In addition, combination therapy consisting of drugs with different mechanisms of action has been recommended to further lower BP without causing adverse drug reactions (ADRs) [2].

Mineralocorticoid receptor antagonists (MRAs) have been shown to exert antihypertensive effects by binding to mineralocorticoid receptors (MRs) and blocking MR-dependent signal transduction. Hyperkalemia is a known major ADR for the MRAs eplerenone and spironolactone [6], as MRAs enhance sodium excretion and potassium reabsorption upon binding to MRs in the renal tubules. Hyperkalemia occurs more frequently after combined treatment with an ARB/ACE inhibitor and an MRA; thus, MRAs should be used carefully, especially when used in combination.

As of March 2016, eplerenone has been approved in over 70 countries (the brand name of eplerenone is Selara in Japan and Inspra® in other countries) for heart failure after acute myocardial infarction and/or heart failure with mild symptoms. However, it has been approved for the treatment of hypertension in only 11 countries including Japan, the United States, Canada, and Singapore. This postmarketing surveillance (PMS) was conducted to identify unknown ADRs that are not stated in the package insert of Selara tablets [7], estimate the incidence of ADRs including hyperkalemia in general practice, and elucidate the factors that affect the safety of the drug when it is used in Japanese hypertensive patients. Additionally, the antihypertensive effects of eplerenone were evaluated.

## 2. Methods

### 2.1. Data Collection and Analysis

Between May 2008 and April 2012, we conducted a PMS that targeted hypertensive patients in Japan who had not previously been treated with eplerenone. The surveillance was conducted in accordance with Articles 14-4 and 14-6 of the Pharmaceutical Affairs Law and in accordance with a protocol approved by the Ministry of Health, Labor, and Welfare (MHLW) of Japan. A written agreement was obtained from participating institutions. The study was also in accordance with the standard of Good Postmarketing Study Practice (GPSP). GPSP is the authorized standard for PMS studies of approved drugs in clinical practice, and no formal ethics committee approval or informed consent was necessary to conduct surveillance under this ordinance.

Because a PMS does not restrict the administration of the study drug or concomitant treatments, the outcomes observed in the PMS reflect the overall consequences of administration of the study drug and concomitant treatments in real-world settings. We aimed to collect 3,000 cases using a centralized registration method to be able to detect unknown ADRs at a frequency of 0.1% with a reliability of 95%.

### 2.2. Patients

Patients were eplerenone-naïve and had essential hypertension diagnosed by physicians in charge. There were no exclusion criteria for patient registration. The physicians in charge were encouraged to consult the Guidelines for the Management of Hypertension 2004 to determine the severity of hypertension in a comprehensive manner. For hepatic function abnormality and renal impairment, categories determined by the physician in charge were adopted. Although precise definitions of severity were not provided, the physicians in charge were asked to judge the severity in a comprehensive manner, considering the disease duration, complications, concomitant medications, and other relevant factors.

### 2.3. Dosage and Administration

In principle, patients received 50–100 mg of eplerenone once a day orally, and the dose was adjusted as necessary. Administration of any concomitant treatment including other antihypertensive drugs was not restricted. The observation period was 12 weeks and began upon initiation of treatment.

### 2.4. Assessment of Safety

Adverse events (AEs) were assessed as a safety endpoint. All unfavorable or unintended signs, symptoms, and diseases that occurred in patients who received eplerenone, regardless of whether there was a clear causal relationship with eplerenone, were considered AEs. If there was a clear causal relationship between eplerenone and an AE, it was considered an ADR. Events were categorized as ADRs when either the physician in charge or the sponsoring company (Pfizer Japan Inc.), or both, determined that the categorization was appropriate. “Hyperkalemia” and “increased serum potassium levels” were categorized as different ADRs based on the terms reported by the physician in charge, because increased serum potassium levels are not necessarily equivalent to hyperkalemia.

A severe ADR was defined as (1) death, (2) a life-threatening event, (3) hospitalization or extension of the hospitalization period, (4) a permanent or remarkable disorder/dysfunction, (5) a congenital abnormality/defect, (6) another medically significant event, or (7) an event that could lead to disability.

Of the patients that were surveyed, it was confirmed that those included in the safety analysis had taken eplerenone at least once during the observation period. However, patients that did not meet the requirements for determining safety according to the selection criteria (i.e., cases involving a breach of contract/imperfect contract and/or registration violations, cases in which target drug use was unable to be confirmed, and cases in which information regarding the presence of AEs was unknown) were excluded from the safety analysis.

### 2.5. Assessments of Effectiveness

The effectiveness of blood pressure reduction was assessed 12 ± 2 weeks after initiation of eplerenone treatment. The population for the effectiveness analysis excluded patients from the safety analysis with contraindicated/off-label use of eplerenone. The exclusion criteria included the following: (1) eplerenone at a dosage of <25 mg or >100 mg per day during the PMS; (2) hyperkalemia (diagnosed by a physician in charge) or high serum potassium levels (>5.0 mEq/L) at baseline; (3) diabetes with microalbuminuria or proteinuria; (4) moderate or severe renal impairment (creatinine clearance rate <50 mL/min); (5) severe hepatic function disorder; and (6) treatment with potassium supplements, potassium-sparing diuretics, itraconazole, ritonavir, or nelfinavir. Creatinine clearance was calculated using the Cockcroft-Gault formula.

### 2.6. Statistics

The use of the terminology for AEs and the classification of events into System Organ Classes were conducted in accordance with the International Conference in Harmonization of Technical Requirements for Registration of Pharmaceuticals for Human Use Medical Dictionary for Regulatory Activities, version 17.1. The number and incidence ((%): (the number of ADR events)/(the number of surveyed cases)) of ADRs were determined by using the System Organ Classes and terminology for AEs. If the severity, treatment, or outcome of an AE was missing, it was considered “unknown.”

In the analysis of the binary data, the frequencies and incidence of AEs were calculated. The 95% two-sided confidence intervals for the incidence were calculated. Fisher's exact test was used to test relationships with nominal data, whereas the Cochran-Armitage test (exact method) was used to test relationships with ordinal data. The data from the analyses were presented (unless otherwise noted) as the mean ± standard deviation or as the percentage of total patients (%). Data processing and analysis were performed using SAS Version 9 (SAS Institute, Cary, NC).

## 3. Results

In the current PMS, 3,374 patients were registered from 400 of 557 contracted institutions. Filled case report forms for 3,318 of these patients were collected from 386 institutions. The participating institutions mainly included private practices and clinics (60.6%). The other institutions included national, public, and private university hospitals. Of the 3,318 cases in which the PMS had been completed, 152 were excluded because of a breach of contract or imperfect contract (*N* = 96), lack of information regarding AEs (no revisit, *N* = 39), violation of registration (*N* = 14), or a lack of information regarding eplerenone treatment (*N* = 3). In total, 3,166 patients were therefore included in the safety analysis. The effect of eplerenone on BP was analyzed in 1,024 patients who met the criteria stated in the method section.

### 3.1. Subject Characteristics

Among the 3,166 patients included in the safety analysis, 51.8% were male, and the mean age was 67.6 ± 12.8 years. Elderly individuals 65 years of age or older accounted for 62.0% of the patients (Table 1). The mean body mass index was 24.4 ± 4.1 kg/m^2^. The severity of hypertension was mild in 29.1%, moderate in 48.9%, and severe in 12.5% of the patients. The mean serum potassium levels were 4.1 ± 0.5 mEq/L at baseline. Concomitant diabetes was observed in 20.0% of the patients, whereas concomitant heart failure was observed in 16.2%. Concomitant renal impairment was observed in 13.5% of the patients, and the mean creatinine clearance rate was 77.6 ± 33.9 mL/min. Patients who received 50 mg of eplerenone at baseline accounted for 74.5% of the total patients, and the mean daily dosage was 45.5 ± 13.7 mg. The mean duration of eplerenone treatment was 128.0 ± 93.5 days, and the median was 106.0 days. At the time eplerenone treatment was initiated, 74.3% of the patients had been treated with other antihypertensive drugs (e.g., CCB, ARB, ACE inhibitor, direct renin inhibitor, thiazide diuretic, loop diuretic, alpha-blocker, or beta-blocker). CCB (49.0%) was the drug most frequently administered concomitantly with eplerenone, followed by ARB (46.6%). Among the patients included in the safety analysis, eplerenone treatment was discontinued in 10.0% of the patients. The reasons for discontinuation were AEs (19.2%), loss to follow-up (18.2%), insufficient clinical effectiveness (16.7%), cure (effective) (15.1%), abnormal clinical laboratory test results (3.8%), death of the patient (3.8%), or other reasons (23.3%). All the deaths were judged as not attributable to eplerenone.

### 3.2. Safety

#### 3.2.1. Adverse Drug Reactions

Among the patients included in the safety analysis, ADRs were observed in 75, and the incidence was 2.4%. The most common ADRs were hyperkalemia (0.6%), dizziness (0.2%), renal impairment (0.2%), and increased serum potassium (0.2%). ADRs that were unpredictable from the package insert of Selara tablets were observed in 11 patients and included renal impairment (*N* = 3), shingles (*N* = 1), acute myeloid leukemia (*N* = 1), lymphadenitis (*N* = 1), loss of appetite (*N* = 1), spasm (*N* = 1), ear fullness (*N* = 1), gastric ulcer (*N* = 1), fecal incontinence (*N* = 1), and photosensitivity reaction (*N* = 1). No clinically significant change in the mean pulse rate was observed. A detailed description of the ADRs is provided in the Supplementary Material available online at http://dx.doi.org/10.1155/2016/5091951.

#### 3.2.2. Serious Adverse Drug Reactions

Among the patients included in the safety analysis, serious ADRs were observed in 0.5% (Table 3). These included hyperkalemia (0.3%), renal impairment (0.1%), increased serum potassium (0.1%), acute myeloid leukemia (0.03%), decreased appetite (0.03%), hepatic function abnormal (0.03%), and drug interactions (0.03%). The outcomes of these ADRs were “unrecovered” for a patient with acute myeloid leukemia and a patient with hepatic function abnormal and “unknown” for a patient with hyperkalemia, whereas the ADRs “disappeared/recovered” or “became less severe” for all the other patients.

#### 3.2.3. Changes in Serum Potassium Levels

There have been concerns regarding a possible increase in serum potassium levels caused by the pharmacological actions of eplerenone. Hyperkalemia or increased serum potassium was observed in 20 and 6 patients, respectively (Table 2). Among these patients, 88.5% were at least 65 years old and 26.9% were at least 80 years old, and concomitant heart failure and diabetes were observed in 34.6% and 26.9% of these patients, respectively. The ADRs were judged serious in 11 of 26 patients (Table 3). Of these patients, 3 had moderate or mild hepatic function disorder, 8 had moderate or mild renal impairment, and 3 had functional class II heart failure according to the New York Heart Association (NYHA) classification system. The ADRs in all of these patients “disappeared/recovered” or “became less severe,” except for 1 patient with an “unknown” outcome. Among the patients whose outcomes were known, 9 halted or withdrew from eplerenone treatment, and 1 did not change the dose.

The incidence of adverse events was 4.7% in patients with renal impairment, which was 2.4-fold higher than that in patients without renal impairment. Of these adverse events, hyperkalemia accounted for 45%.

Serum potassium levels during the surveillance period slightly increased in patients who had serum potassium levels ≤ 4.5 mEq/L (Table 4) at baseline. The incidence of potassium increase in the patients with baseline serum potassium levels ≤ 3.5 mEq/L was 21.6%, which was higher than that of patients with serum potassium levels over 3.5 mEq/L to 4.5 mEq/L at baseline (6.4%). Very little change in the serum potassium level was observed in patients with serum potassium levels of over 4.5 to 5.0 mEq/L at baseline, whereas the levels slightly decreased in those with serum potassium levels over 5.0 mEq/L at baseline. In contrast, the percentage of patients with serum potassium values over 5.5 mEq/L increased as the baseline serum potassium levels increased. One case of hyperkalemia was observed among the 158 patients who received 100 mg of eplerenone, which was the maximum approved dosage per day, on at least 1 day during the surveillance period. The hyperkalemia disappeared/recovered without changing of the dose in the patient. Importantly, eplerenone is contraindicated in patients with serum potassium levels > 5.5 mEq/L according to the package insert of Selara tablets [7].

#### 3.2.4. Adverse Drug Reactions in Patients with Lower Creatinine Clearance

There were 6 cases that had ADRs with CCr < 30 mL/min at baseline, and the incidence of any ADR was 6.9%. The major ADRs of these patients were hyperkalemia (3.4%) and renal impairment (2.3%). The incidence of ADRs in patients with baseline CCr from greater than or equal to 30 mL/min to less than 50 mL/min was 4.1%, and their major ADRs were also hyperkalemia (1.2%) and increased serum potassium (0.9%). In contrast, the incidence of ADRs in patients with baseline CCr from greater than or equal to 50 mL/min up to 80 mL/min was 2.5%, and major ADRs were also hyperkalemia and increased serum potassium (both 0.4%). It is important to note that eplerenone is contraindicated in patients with CCr < 50 mL/min according to the package insert of Selara tablets.

#### 3.2.5. Adverse Drug Reactions in Patients Prescribed Other Antihypertensive Drugs

Among the 2,355 patients who were prescribed antihypertensive medications other than eplerenone at the time of initiation of eplerenone treatment, 2 cases developed hypotension. Both were prescribed more than 2 antihypertensive drugs in addition to eplerenone. The incidence of hyperkalemia among patients prescribed ACE inhibitors or ARBs concomitantly with eplerenone was 1.1% and 0.8%, respectively, and that of patients without concomitant use of ACE inhibitors or ARBs was 0.6% and 0.5%, respectively. The incidence of hyperkalemia among patients prescribed thiazide diuretics or loop diuretics together with eplerenone was 0% and 0.9%, respectively, and the incidence in patients treated without these drugs was 0.7% and 0.6%, respectively. The incidence of adverse drug reactions classified according to the concomitant antihypertensive medication is presented in the Supplementary Material (Table S2 to S9). Serious adverse events were seen in 15 among 2,355 patients who were taking concomitant antihypertensive medications at the time of initiation of eplerenone. The most frequent serious adverse events were hyperkalemia (8 cases) followed by renal impairment (3 cases).

### 3.3. Effectiveness

#### 3.3.1. Blood Pressure Control

**Figure 1:**
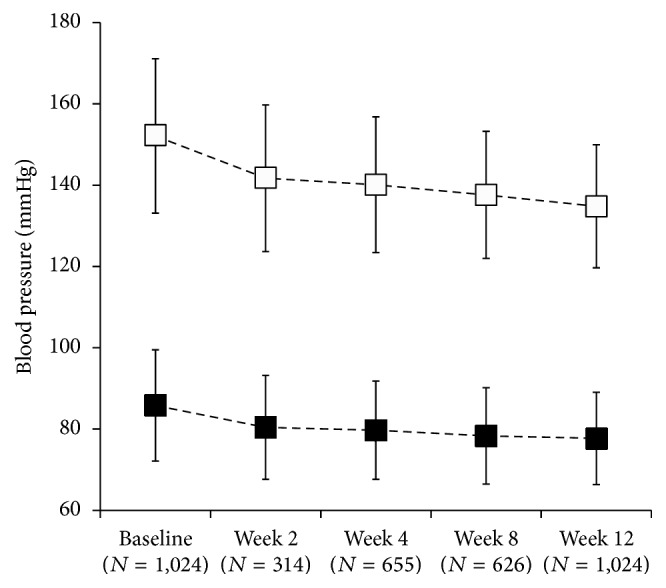
Changes in systolic (open square) and diastolic (closed square) blood pressure at each observation time point from baseline to week 12 (mean ± standard deviation) during the 12-week treatment period in the effectiveness analysis population.

**Figure 2:**
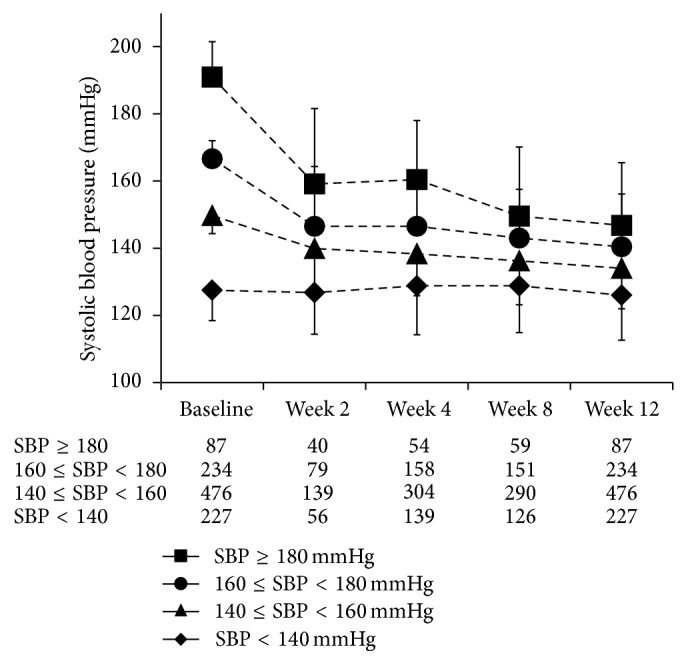
Changes in systolic blood pressure (mean ± standard deviation) from baseline to each observation time point during the 12-week treatment period in the effectiveness analysis population. The number of patients at each time point is indicated by the SBP levels under the chart.

Of the 1,024 patients for whom the effect of eplerenone on BP was analyzed, the mean SBP and the mean DBP at baseline were 152.1 ± 19.0 mmHg and 85.8 ± 13.7 mmHg, respectively (Figure 1). The changes in mean SBP and DBP from baseline to week 12 were −17.3 ± 18.4 mmHg (percent change from baseline: −10.6 ± 11.4%) and −8.1 ± 11.8 mmHg (−8.3 ± 13.4%), respectively. The changes in mean SBP and DBP from baseline to week 2 were −13.5 ± 18.1 and −6.7 ± 11.1 mmHg, respectively. The changes in SBP from baseline to week 12 in < 140 mmHg, 140 mmHg to < 160 mmHg, 160 mmHg to < 180 mmHg, and ≥ 180 mmHg groups were the following: −1.5 ± 14.7 mmHg, −15.6 ± 12.0 mmHg, −26.3 ± 15.5 mmHg, and −44.1 ± 19.6 mmHg (Figure 2). The BP changes from baseline were larger in the groups with higher baseline BP, and there was little change in the group with the lowest BP level at baseline.

## 4. Discussion

### 4.1. Subjects Included in the Analysis

Among the patients registered in this PMS, patients over 55 years of age accounted for approximately 80%, which is similar to the fraction of elderly hypertensive patients in Japan [2]. In this surveillance, more than 70% of the patients had been prescribed at least one antihypertensive drug at the time that eplerenone treatment was initiated. MRAs are not the first-line drugs recommended by the JSH 2009 or JSH 2014 guidelines [8], but they are recommended for treatment of resistant hypertension. This PMS showed that CCBs and ARBs, which are recommended as first-line drugs by the guidelines, were the most frequent drugs to be prescribed concomitantly with eplerenone.

### 4.2. Safety Analysis

In the current PMS, the most common ADRs were hyperkalemia (0.6%), dizziness (0.2%), renal impairment (0.2%), and increased serum potassium (0.2%). The ADRs most frequently observed in the clinical trials cited in the new-drug application of eplerenone in Japan included headache (6.1%), dizziness (2.6%), nausea (1.9%), hyperkalemia (1.7%), fatigue (1.6%), increased alanine aminotransferase (1.4%), increased gamma-glutamyl transferase (1.3%), indigestion (1.2%), increased aspartate aminotransferase (1.2%), muscle spasms (1.0%), and hyperuricemia (1.0%). The types of ADRs observed in this PMS were similar to those observed in the clinical trials, and no notable new ADRs were observed. The difference in the frequency of ADRs between the PMS and the clinical trials is attributable to differences in study design, as a PMS is an observational study and the clinical trials were interventional studies.

According to a survey by the Japanese Society of Nephrology [9], the frequency of CKD increases with age among Japanese men and women. In particular, most patients had stage 3 CKD (glomerular filtration rate: 30–59 mL/min/1.73 m^2^): 15.6% of men in their 60s and 43.1% of men over 80 years of age. In this PMS, patients who were reported to have hyperkalemia or increased serum potassium accounted for 88.5% of patients aged 65 years or over, and 26.9% of patients aged 80 years or over. Retention of serum potassium is known to occur more frequently in patients with renal impairment. In this PMS, the occurrence of adverse events was more frequent in patients with renal impairment, and many of the events were hyperkalemia. Therefore, the results of this surveillance also suggest that patients with renal impairment are more prone to hyperkalemia and that serum potassium levels should be properly monitored according to the package insert. Regarding the change in serum potassium levels during the treatment period, it is possible that the physicians adjusted the eplerenone dosage or withdrew other potassium-retaining drugs that had been administered concomitantly in patients with high baseline serum potassium levels.

In this PMS, some patients were prescribed eplerenone even though they met the contraindication criteria for Selara tablets. Patients with a serum potassium value over 5.0 mEq/L comprised 1.9% of the safety analysis population. There were 14.8% patients whose CCr was less than 50 mL/min calculated from their sex, age, and weight by Pfizer Japan Inc. or judged by the physicians as having as moderate or severe renal impairment. In addition, patients with severe hepatic function comprised 0.2% of the safety analysis population. Clinicians should pay careful attention to the contraindications listed on the package insert when prescribing drugs and conducting regular monitoring of serum potassium levels.

The incidence of hypotension among patients prescribed antihypertensive drugs in addition to eplerenone was considered as not high and the symptoms were improved. Although the incidence of hyperkalemia tended to be higher among patients prescribed ACE inhibitors or ARBs concomitantly with eplerenone, it was considered within the scope of the package insert of Selara tablets. There was no tendency for the incidence of hyperkalemia to increase when eplerenone was used with either thiazide diuretics or loop diuretics. Therefore, we concluded that it is not necessary to call for special attention or implement new measures to ensure safety for concomitant use of eplerenone and other antihypertensive drugs.

### 4.3. Antihypertensive Effects

In an analysis of the antihypertensive effects of eplerenone based on the BP at baseline, the degree of BP reduction appeared to vary depending on the BP value at the time of initiation of eplerenone. Because we aimed to investigate the effects of the drugs in real-world settings and because the dosage of all of the antihypertensive drugs including eplerenone could have been modified, the results do not necessarily reflect the effects of eplerenone treatment alone.

However, considering that, on average, clinically significant effectiveness against hypertension was observed during the observation period, the effectiveness of eplerenone as an antihypertensive drug was shown under the conditions in which it is currently used.

Because a relatively large antihypertensive effect was observed immediately after initiation of eplerenone treatment followed by a gradual decrease in BP, a decrease of several mmHg after week 12 was expected. The finding that eplerenone gradually reduced BP over 2-3 months is consistent with a previous report [9]. The reason for the gradual decrease in BP and the antihypertensive effects of eplerenone may involve the complex mechanism of action of the drug. Aldosterone, a ligand for MRs, is a hormone involved in the reabsorption of sodium by the kidneys. However, recent studies have demonstrated that MRs are expressed in various organs including vascular endothelial and smooth muscle cells found in all cardiovascular tissues [10–14]. Therefore, various factors might contribute to BP reduction through MR blockade.

### 4.4. Organ Damage with Aldosterone

Recent studies have revealed that organ damage occurs in the cardiovascular system and kidneys when aldosterone coexists with high sodium. This organ damage includes ventricular remodeling [15, 16], renal impairment [17–19], and vascular endothelial dysfunction [20]. Furthermore, aldosterone, which exerts its activity in adipocytes, is involved in abnormal production/secretion of adipocytokines by adipocytes [21] and contributes to increases in oxidative stress [22] and decreases insulin sensitivity in adipose tissue [23, 24]. Eplerenone is thought to suppress the adverse effects of aldosterone in in various organs including the endothelium by inhibiting aldosterone activity through binding to MRs [25]; thus, eplerenone is a pharmacologically promising drug to prevent end organ damage induced by aldosterone.

### 4.5. Study Limitations

This surveillance has several limitations. First, PMSs including this study are not required to monitor the data consistency between the medical record and the data reported to the pharmaceutical company, although the data consistency within the case report forms is systematically confirmed. Second, as stated above, because the PMS does not restrict the administration of eplerenone or other antihypertensive drugs, the outcomes observed in this surveillance would reflect the overall consequences of antihypertensive treatments in real-world settings; thus, the treatment could have been changed based on observed AEs or change in blood pressure during the surveillance. Third, because there were no exclusion criteria in this study, patients with severe conditions such as active malignancy were not excluded, which might have affected the safety and efficacy parameters. Fourth, this PMS was conducted as a noninterventional study under real-world settings and the results should be interpreted within this context. For example, potassium concentration data were unavailable (unknown) in 877 subjects, which comprised 27.7% of the safety analysis population (Table 1), because the clinical data could only be collected when the lab tests were conducted in the usual care settings. Fifth, because the method for measuring BP was not standardized, that is, the measuring device and method varied at each institution, it is possible that the assessment of antihypertensive effects varied. However, even if this potential variability is taken into account, our results still showed a decrease in the mean SBP/DBP (17.3/8.1 mmHg); thus, the conclusion of a significant clinical difference would not be affected. Sixth, although this PMS was conducted in patients with essential hypertension, patients with secondary hypertension such as primary aldosteronism (PA) may have also been included without a definite diagnosis. Because PA responds well to MRAs, inclusion of such patients might have contributed to a reduction in the mean BP to some extent. However, as the prevalence of PA is only approximately 10% among patients with essential hypertension [26], the influence on the mean BP would not be substantial. Despite these limitations, this PMS has provided important information regarding the effect of eplerenone in patients with essential hypertension in real-world settings.

## 5. Conclusions

In this PMS, patients treated with eplerenone showed a marked blood pressure reduction over 12 weeks after initiation of treatment. There was no significant difference in the type or incidence of ADRs in this surveillance compared to results reported prior to approval of the drug. With respect to hyperkalemia, the current PMS confirmed that it is important to adhere to the contraindications stated on the package insert. Eplerenone is contraindicated in patients with CCr < 50 mL/min; administration should be very carefully conducted in the elderly. Because recent studies have revealed that aldosterone can contribute to organ damage (e.g., damage to the heart and kidneys), the use of a selective MRA such as eplerenone can provide benefit for patients who need antihypertensive treatment.

## Supplementary Material

This Supplementary Material has been provided by the authors to give readers additional information about their work. The incidence of all and serious adverse drug reactions in safety analysis population (N = 3,166) is provided. The incidence of adverse drug reactions by concomitant antihypertensive medications at the time of initiation of eplerenone in safety analysis population (N = 3,166) are also provided.

## Figures and Tables

**Table 1:** Patient characteristics.

Factors	Categories	Analysis populations
		Safety analysis population, *N* = 3,166 (%)
Sex	Male	1,640 (51.8)

Age (years)	Mean ± SD	67.6 ± 12.8
	<35	21 (0.7)
	35 to <45	149 (4.7)
	45 to <55	343 (10.8)
	55 to <65	689 (21.8)
	65 to <75	897 (28.3)
	75 to <85	845 (26.7)
	≥85	222 (7.0)

BMI (kg/m^2^)	Mean ± SD	24.4 ± 4.1
	<25	1,411 (44.6)
	≥25	875 (27.6)
	Unknown	880 (27.8)

Targeted disease	Hypertension	2,919 (92.2)
	Other reasons	247 (7.8)

Severity of hypertension	Mild	922 (29.1)
	Moderate	1,548 (48.9)
	Severe	395 (12.5)
	Unknown	54 (1.7)

Inpatients/outpatients	Inpatients	191 (6.0)
	Outpatients	2,975 (94.6)

Fasting glucose (mg/dL)	Mean ± SD	110.0 ± 36.8
	<110	1,271 (40.1)
	110 to <126	255 (8.1)
	≥126	294 (9.3)
	Not examined	1,346 (42.5)

HbA1c (%)	Mean ± SD	5.7 ± 1.0
	<5.8	1,106 (34.9)
	5.8 to <6.5	289 (9.1)
	≥6.5	247 (7.8)
	Not examined	1,524 (48.1)

Hepatic function disorder	No	2,647 (83.6)
	Yes	472 (14.9)
	Mild	405 (12.8)
	Moderate	47 (1.5)
	Severe	6 (0.2)
	Unknown	14 (0.4)
	Unknown	47 (1.5)

Renal impairment	No	2,697 (85.2)
	Yes	426 (13.5)
	Mild	323 (10.2)
	Moderate	88 (2.8)
	Severe	11 (0.3)
	Unknown	4 (0.1)
	Unknown	43 (1.4)

Heart failure	No	2,631 (83.1)
	Yes	513 (16.2)
	Unknown	22 (0.7)

NYHA functional class	I	197 (6.2)
	II	199 (6.3)
	III	56 (1.8)
	IV	13 (0.4)
	Unknown	70 (2.2)

Diabetes	No	2,490 (78.6)
	Yes	634 (20.0)
	Unknown	42 (1.3)

Primary aldosteronism	No	3,121 (98.6)
	Yes	45 (1.4)

Initial dose of eplerenone (mg/day)	Mean ± SD	45.5 ± 13.7
	25	718 (22.7)
	50	2,358 (74.5)
	75	6 (0.2)
	100	78 (2.5)
	Other reasons	6 (0.2)

Serum potassium concentrations (mEq/L)	Mean ± SD	4.1 ± 0.5
	≤3.5	239 (7.5)
	>3.5 to ≤5.0	1,991 (62.9)
	>5.0	59 (1.9)
	Unknown	877 (27.7)

Creatinine clearance (mL/min)	Mean ± SD	77.6 ± 33.9
	>80	851 (26.9)
	≥50 to ≤80	753 (23.8)
	≥30 to <50	345 (10.9)
	<30	87 (2.7)
	Unknown	1,130 (35.7)

SD: standard deviation; BMI: body mass index; HbA1c: glycated hemoglobin A1c; NYHA: New York Heart Association; CrCl: creatinine clearance.

**Table 2:** Incidence of adverse drug reactions in safety analysis population.

Adverse drug reactions: SOC	*N* (%)	Major types: PT (occurrence ≥ 0.1%) (*N* (%))
*Number of patients in safety analysis population*	3,166 (100)	

*Patients with adverse drug reactions*	75 (2.37)	
*Total number of adverse drug reactions*	82	
Infections and infestations	1 (0.03)	
Neoplasms (benign, malignant, and unspecified, including cysts and polyps)	1 (0.03)	
Blood and lymphatic system disorders	1 (0.03)	
Metabolism and nutrition disorders	25 (0.79)	Hyperkalemia: 20 (0.63)
Nervous system disorders	10 (0.32)	Dizziness: 7 (0.22)
Ear and labyrinth disorders	1 (0.03)	
Cardiac disorders	1 (0.03)	
Vascular disorders	3 (0.09)	
Respiratory, thoracic, and mediastinal disorders	1 (0.03)	
Gastrointestinal disorders	6 (0.19)	
Hepatobiliary disorders	2 (0.06)	
Skin and subcutaneous tissue disorders	5 (0.16)	
Renal and urinary disorders	10 (0.32)	Renal impairment: 7 (0.22)
General disorders and administration site conditions	4 (0.13)	
Investigations	11 (0.35)	Increased serum potassium: 6 (0.19)

Calculated with MedDRA/J17.1 System Organ Class and preferred terms.

**Table 3:** Incidence of serious adverse drug reactions.

Serious adverse drug reactions	*N* (%)
Safety analysis population	3,166
Patients with adverse drug reactions	16 (0.51)
*Neoplasms (benign, malignant, and unspecified, including cysts and polyps)*	*1 (0.03)*
Acute myeloid leukemia	1 (0.03)
*Metabolism and nutrition disorders*	*10 (0.32)*
Hyperkalemia	9 (0.28)
Decreased appetite	1 (0.03)
*Hepatobiliary disorders*	*1 (0.03)*
Hepatic function abnormal	1 (0.03)
*Renal and urinary disorders*	*3 (0.09)*
Renal impairment	3 (0.09)
*General disorders and administration site conditions*	*1 (0.03)*
Drug interaction	1 (0.03)
*Investigations*	*2 (0.06)*
Blood potassium increased	2 (0.06)

Calculated with MedDRA/J17.1 System Organ Class and preferred terms.

**Table 4:** Serum potassium levels during eplerenone treatment relative to baseline levels.

Baseline level (mEq/L)	*N*	Maximal value (mEq/L)	Changes from baseline (mEq/L)	% changes from baseline (%)	Patients with ≥5.5 mEq/L (*N* (% [95% CI]))
Serum potassium ≤ 3.5	195	3.9 ± 0.4	0.69 ± 0.45	21.6 ± 15.5	0 (0.0 [0.0–1.9])
3.5 < serum potassium ≤ 4.5	1315	4.3 ± 0.4	0.25 ± 0.42	6.4 ± 10.6	19 (1.4 [0.9–2.2])
4.5 < serum potassium ≤ 5.0	263	4.7 ± 0.5	−0.02 ± 0.51	−0.27 ± 10.7	14 (5.3 [2.9–8.8])
Serum potassium > 5.0	51	5.0 ± 0.6	−0.41 ± 0.80	−7.1 ± 13.7	10 (19.6 [9.8–33.1])

Mean ± standard deviation. CI: confidence interval. The cases who had serum potassium data at baseline and additional timings during the PMS were included in this table. The number of the cases was 1,824 out of the safety analysis population (*N* = 3,166).
