# Supplementary material for: Safety and Antihypertensive Effect of Selara® (Eplerenone): Results from a Postmarketing Surveillance in Japan
Source: Int J Hypertens. 2016 Oct 24;2016:5091951. doi: 10.1155/2016/5091951 (PMC5098080; doi:10.1155/2016/5091951)
Supplement: Supplementary file 1 — This Supplementary Material has been provided by the authors to give readers additional information about their work. The incidence of all and serious adverse drug reactions in safety analysis population (N = 3,166) is provided. The incidence of adverse drug reactions by concomitant antihypertensive medications at the time of initiation of eplerenone in safety analysis population (N = 3,166) are also provided. [file 5091951.f1.docx]

Supplemental data

Table S1. Incidence of all and serious adverse drug reactions in safety analysis population (N = 3,166)

| Adverse drug reactions | All (N (%)) | Serious  (N (%)) |
| --- | --- | --- |
| Patients with adverse drug reactions | 75 (2.37) | 16 (0.51) |
| *Infections and infestations* | *1 (0.03)* | *0* |
| *Herpes zoster* | 1 (0.03) | 0 |
| *Neoplasms (benign, malignant, and unspecified, including cysts and polyps)* | *1 (0.03)* | *1 (0.03)* |
| Acute myeloid leukemia | 1 (0.03) | 1 (0.03) |
| *Blood and lymphatic system disorders* | *1 (0.03)* | *0* |
| Lymphadenitis | 1 (0.03) | 0 |
| *Metabolism and nutrition disorders* | *25 (0.79)* | *10 (0.32)* |
| Hyperkalemia | 20 (0.63) | 9 (0.28) |
| Hyperuricemia | 1 (0.03) | 0 |
| Decreased appetite | 1 (0.03) | 1 (0.03) |
| Dehydration | 1 (0.03) | 0 |
| Hyponatremia | 2 (0.06) | 0 |
| *Nervous system disorders* | *10 (0.32)* | *0* |
| Hypoesthesia | 1 (0.03) | 0 |
| Headache | 1 (0.03) | 0 |
| Dizziness | 7 (0.22) | 0 |
| Convulsion | 1 (0.03) | 0 |
| *Ear and labyrinth disorders* | *1 (0.03)* | *0* |
| Ear congestion | 1 (0.03) | 0 |
| *Cardiac disorders* | *1 (0.03)* | *0* |
| Palpitations | 1 (0.03) | 0 |
| *Vascular disorders* | *3 (0.09)* | *0* |
| Orthostatic hypotension | 1 (0.03) | 0 |
| Hypotension | 2 (0.06) | 0 |
| *Respiratory, thoracic and mediastinal disorders* | *1 (0.03)* | *0* |
| Pharyngeal edema | 1 (0.03) | 0 |
| *Gastrointestinal disorders* | *6 (0.19)* | *0* |
| Nausea | 3 (0.09) | 0 |
| Gastric ulcer | 1 (0.03) | 0 |
| Diarrhea | 1 (0.03) | 0 |
| Fecal incontinence | 1 (0.03) | 0 |
| *Hepatobiliary disorders* | *2 (0.06)* | *1 (0.03)* |
| Hepatic function abnormal | 1 (0.03) | 1 (0.03) |
| Liver disorder | 1 (0.03) | 0 |
| *Skin and subcutaneous tissue disorders* | *5 (0.16)* | *0* |
| Photosensitivity reaction | 1 (0.03) | 0 |
| Rash | 3 (0.09) | 0 |
| Drug eruption | 1 (0.03) | 0 |
| *Renal and urinary disorders* | *10 (0.32)* | *3 (0.09)* |
| Renal impairment | 7 (0.22) | 3 (0.09) |
| Polyuria | 1 (0.03) | 0 |
| Frequent urination | 1 (0.03) | 0 |
| Nocturia | 1 (0.03) | 0 |
| *General disorders and administration site conditions* | *4 (0.13)* | *1 (0.03)* |
| Malaise | 1 (0.03) | 0 |
| Edema | 1 (0.03) | 0 |
| Peripheral swelling | 1 (0.03) | 0 |
| Drug interaction | 1 (0.03) | 1 (0.03) |
| *Investigations* | *11 (0.35)* | *2 (0.06)* |
| Blood pressure decreased | 1 (0.03) | 0 |
| Blood potassium increased | 6 (0.19) | 2 (0.06) |
| Blood creatinine increased | 2 (0.06) | 0 |
| Blood cholesterol increased | 1 (0.03) | 0 |
| Blood urea increased | 1 (0.03) | 0 |

Calculated with MedDRA/J17.1　System Organ Class and Preferred Terms.

Table S2. Incidence of adverse drug reactions by concomitant antihypertensive medication (calcium channel blocker) at the time of initiation of eplerenone in safety analysis population (N = 3,166)

| Adverse drug reactions | Concomitant antihypertensive medication | | | | | |
| --- | --- | --- | --- | --- | --- | --- |
|  | with CCB (N (%)) | | without CCB (N (%)) | | Unknown (N (%)) | |
| Safety analysis population | 1551 | | 1615 | | 0 | |
| Patients with adverse drug reactions | 40 | (2.58) | 35 | (2.17) | - |  |
| *Infections and infestations* | 1 | (0.06) | 0 | (0.00) | - |  |
| *Herpes zoster* | 1 | (0.06) | 0 | (0.00) | - |  |
| *Neoplasms (benign, malignant, and unspecified, including cysts and polyps)* | 1 | (0.06) | 0 | (0.00) | - |  |
| Acute myeloid leukemia | 1 | (0.06) | 0 | (0.00) | - |  |
| *Blood and lymphatic system disorders* | 1 | (0.06) | 0 | (0.00) | - |  |
| Lymphadenitis | 1 | (0.06) | 0 | (0.00) | - |  |
| *Metabolism and nutrition disorders* | 14 | (0.90) | 11 | (0.68) | - |  |
| Hyperkalemia | 10 | (0.64) | 10 | (0.62) | - |  |
| Hyperuricemia | 1 | (0.06) | 0 | (0.00) | - |  |
| Decreased appetite | 1 | (0.06) | 0 | (0.00) | - |  |
| Dehydration | 1 | (0.06) | 0 | (0.00) | - |  |
| Hyponatremia | 1 | (0.06) | 1 | (0.06) | - |  |
| *Nervous system disorders* | 5 | (0.32) | 5 | (0.31) | - |  |
| Hypoesthesia | 0 | (0.00) | 1 | (0.06) | - |  |
| Headache | 1 | (0.06) | 0 | (0.00) | - |  |
| Dizziness | 3 | (0.19) | 4 | (0.25) | - |  |
| Convulsion | 1 | (0.06) | 0 | (0.00) | - |  |
| *Ear and labyrinth disorders* | 0 | (0.00) | 1 | (0.06) | - |  |
| Ear congestion | 0 | (0.00) | 1 | (0.06) | - |  |
| *Cardiac disorders* | 0 | (0.00) | 1 | (0.06) | - |  |
| Palpitations | 0 | (0.00) | 1 | (0.06) | - |  |
| *Vascular disorders* | 0 | (0.00) | 3 | (0.19) | - |  |
| Orthostatic hypotension | 0 | (0.00) | 1 | (0.06) | - |  |
| Hypotension | 0 | (0.00) | 2 | (0.12) | - |  |
| *Respiratory, thoracic and mediastinal disorders* | 0 | (0.00) | 1 | (0.06) | - |  |
| Pharyngeal edema | 0 | (0.00) | 1 | (0.06) | - |  |
| *Gastrointestinal disorders* | 4 | (0.26) | 2 | (0.12) | - |  |
| Nausea | 2 | (0.13) | 1 | (0.06) | - |  |
| Gastric ulcer | 1 | (0.06) | 0 | (0.00) | - |  |
| Diarrhea | 0 | (0.00) | 1 | (0.06) | - |  |
| Fecal incontinence | 1 | (0.06) | 0 | (0.00) | - |  |
| *Hepatobiliary disorders* | 1 | (0.06) | 1 | (0.06) | - |  |
| Hepatic function abnormal | 1 | (0.06) | 0 | (0.00) | - |  |
| Liver disorder | 0 | (0.00) | 1 | (0.06) | - |  |
| *Skin and subcutaneous tissue disorders* | 2 | (0.13) | 3 | (0.19) | - |  |
| Photosensitivity reaction | 0 | (0.00) | 1 | (0.06) | - |  |
| Rash | 1 | (0.06) | 2 | (0.12) | - |  |
| Drug eruption | 1 | (0.06) | 0 | (0.00) | - |  |
| *Renal and urinary disorders* | 7 | (0.45) | 3 | (0.19) | - |  |
| Renal impairment | 5 | (0.32) | 2 | (0.12) | - |  |
| Polyuria | 1 | (0.06) | 0 | (0.00) | - |  |
| Frequent urination | 0 | (0.00) | 1 | (0.06) | - |  |
| Nocturia | 1 | (0.06) | 0 | (0.00) | - |  |
| *General disorders and administration site conditions* | 2 | (0.13) | 2 | (0.12) | - |  |
| Malaise | 1 | (0.06) | 0 | (0.00) | - |  |
| Edema | 0 | (0.00) | 1 | (0.06) | - |  |
| Peripheral swelling | 0 | (0.00) | 1 | (0.06) | - |  |
| Drug interaction | 1 | (0.06) | 0 | (0.00) | - |  |
| *Investigations* | 7 | (0.45) | 4 | (0.25) | - |  |
| Blood pressure decreased | 1 | (0.06) | 0 | (0.00) | - |  |
| Blood potassium increased | 5 | (0.32) | 1 | (0.06) | - |  |
| Blood creatinine increased | 0 | (0.00) | 2 | (0.12) | - |  |
| Blood cholesterol increased | 0 | (0.00) | 1 | (0.06) | - |  |
| Blood urea increased | 1 | (0.06) | 0 | (0.00) | - |  |

Calculated with MedDRA/J17.1　System Organ Class and Preferred Terms. CCB: calcium channel blocker

Table S3. Incidence of adverse drug reactions by concomitant antihypertensive medication (alpha blocker) at the time of initiation of eplerenone in safety analysis population (N = 3,166)

| Adverse drug reactions | Concomitant antihypertensive medication | | | | | |
| --- | --- | --- | --- | --- | --- | --- |
|  | With alpha blocker (N (%)) | | Without alpha blocker (N (%)) | | Unknown (N (%)) | |
| Safety analysis population | 416 | | 2750 | | 0 | |
| Patients with adverse drug reactions | 11 | (2.64) | 64 | (2.33) | - |  |
| *Infections and infestations* | 0 | (0.00) | 1 | (0.04) | - |  |
| *Herpes zoster* | 0 | (0.00) | 1 | (0.04) | - |  |
| *Neoplasms (benign, malignant, and unspecified, including cysts and polyps)* | 0 | (0.00) | 1 | (0.04) | - |  |
| Acute myeloid leukemia | 0 | (0.00) | 1 | (0.04) | - |  |
| *Blood and lymphatic system disorders* | 0 | (0.00) | 1 | (0.04) | - |  |
| Lymphadenitis | 0 | (0.00) | 1 | (0.04) | - |  |
| *Metabolism and nutrition disorders* | 3 | (0.72) | 22 | (0.80) | - |  |
| Hyperkalemia | 3 | (0.72) | 17 | (0.62) | - |  |
| Hyperuricemia | 0 | (0.00) | 1 | (0.04) | - |  |
| Decreased appetite | 0 | (0.00) | 1 | (0.04) | - |  |
| Dehydration | 0 | (0.00) | 1 | (0.04) | - |  |
| Hyponatremia | 0 | (0.00) | 2 | (0.07) | - |  |
| *Nervous system disorders* | 0 | (0.00) | 10 | (0.36) | - |  |
| Hypoesthesia | 0 | (0.00) | 1 | (0.04) | - |  |
| Headache | 0 | (0.00) | 1 | (0.04) | - |  |
| Dizziness | 0 | (0.00) | 7 | (0.25) | - |  |
| Convulsion | 0 | (0.00) | 1 | (0.04) | - |  |
| *Ear and labyrinth disorders* | 0 | (0.00) | 1 | (0.04) | - |  |
| Ear congestion | 0 | (0.00) | 1 | (0.04) | - |  |
| *Cardiac disorders* | 1 | (0.24) | 0 | (0.00) | - |  |
| Palpitations | 1 | (0.24) | 0 | (0.00) | - |  |
| *Vascular disorders* | 1 | (0.24) | 2 | (0.07) | - |  |
| Orthostatic hypotension | 0 | (0.00) | 1 | (0.04) | - |  |
| Hypotension | 1 | (0.24) | 1 | (0.04) | - |  |
| *Respiratory, thoracic and mediastinal disorders* | 0 | (0.00) | 1 | (0.04) | - |  |
| Pharyngeal edema | 0 | (0.00) | 1 | (0.04) | - |  |
| *Gastrointestinal disorders* | 2 | (0.48) | 4 | (0.15) | - |  |
| Nausea | 1 | (0.24) | 2 | (0.07) | - |  |
| Gastric ulcer | 0 | (0.00) | 1 | (0.04) | - |  |
| Diarrhea | 0 | (0.00) | 1 | (0.04) | - |  |
| Fecal incontinence | 1 | (0.24) | 0 | (0.00) | - |  |
| *Hepatobiliary disorders* | 0 | (0.00) | 2 | (0.07) | - |  |
| Hepatic function abnormal | 0 | (0.00) | 1 | (0.04) | - |  |
| Liver disorder | 0 | (0.00) | 1 | (0.04) | - |  |
| *Skin and subcutaneous tissue disorders* | 0 | (0.00) | 5 | (0.18) | - |  |
| Photosensitivity reaction | 0 | (0.00) | 1 | (0.04) | - |  |
| Rash | 0 | (0.00) | 3 | (0.11) | - |  |
| Drug eruption | 0 | (0.00) | 1 | (0.04) | - |  |
| *Renal and urinary disorders* | 2 | (0.48) | 8 | (0.29) | - |  |
| Renal impairment | 2 | (0.48) | 5 | (0.18) | - |  |
| Polyuria | 0 | (0.00) | 1 | (0.04) | - |  |
| Frequent urination | 0 | (0.00) | 1 | (0.04) | - |  |
| Nocturia | 0 | (0.00) | 1 | (0.04) | - |  |
| *General disorders and administration site conditions* | 0 | (0.00) | 4 | (0.15) | - |  |
| Malaise | 0 | (0.00) | 1 | (0.04) | - |  |
| Edema | 0 | (0.00) | 1 | (0.04) | - |  |
| Peripheral swelling | 0 | (0.00) | 1 | (0.04) | - |  |
| Drug interaction | 0 | (0.00) | 1 | (0.04) | - |  |
| *Investigations* | 2 | (0.48) | 9 | (0.33) | - |  |
| Blood pressure decreased | 0 | (0.00) | 1 | (0.04) | - |  |
| Blood potassium increased | 2 | (0.48) | 4 | (0.15) | - |  |
| Blood creatinine increased | 0 | (0.00) | 2 | (0.07) | - |  |
| Blood cholesterol increased | 0 | (0.00) | 1 | (0.04) | - |  |
| Blood urea increased | 0 | (0.00) | 1 | (0.04) | - |  |

Calculated with MedDRA/J17.1　System Organ Class and Preferred Terms.

Table S4. Incidence of adverse drug reactions by concomitant antihypertensive medication (beta blocker) at the time of initiation of eplerenone in safety analysis population (N = 3,166)

| Adverse drug reactions | Concomitant antihypertensive medication | | | | | |
| --- | --- | --- | --- | --- | --- | --- |
|  | With beta blocker (N (%)) | | Without beta blocker (N (%)) | | Unknown (N (%)) | |
| Safety analysis population | 535 | | 2631 | | 0 | |
| Patients with adverse drug reactions | 16 | (2.99) | 59 | (2.24) | - |  |
| *Infections and infestations* | 0 | (0.00) | 1 | (0.04) | - |  |
| *Herpes zoster* | 0 | (0.00) | 1 | (0.04) | - |  |
| *Neoplasms (benign, malignant, and unspecified, including cysts and polyps)* | 0 | (0.00) | 1 | (0.04) | - |  |
| Acute myeloid leukemia | 0 | (0.00) | 1 | (0.04) | - |  |
| *Blood and lymphatic system disorders* | 0 | (0.00) | 1 | (0.04) | - |  |
| Lymphadenitis | 0 | (0.00) | 1 | (0.04) | - |  |
| *Metabolism and nutrition disorders* | 4 | (0.75) | 21 | (0.80) | - |  |
| Hyperkalemia | 4 | (0.75) | 16 | (0.61) | - |  |
| Hyperuricemia | 0 | (0.00) | 1 | (0.04) | - |  |
| Decreased appetite | 0 | (0.00) | 1 | (0.04) | - |  |
| Dehydration | 0 | (0.00) | 1 | (0.04) | - |  |
| Hyponatremia | 0 | (0.00) | 2 | (0.08) | - |  |
| *Nervous system disorders* | 1 | (0.19) | 9 | (0.34) | - |  |
| Hypoesthesia | 0 | (0.00) | 1 | (0.04) | - |  |
| Headache | 0 | (0.00) | 1 | (0.04) | - |  |
| Dizziness | 1 | (0.19) | 6 | (0.23) | - |  |
| Convulsion | 0 | (0.00) | 1 | (0.04) | - |  |
| *Ear and labyrinth disorders* | 0 | (0.00) | 1 | (0.04) | - |  |
| Ear congestion | 0 | (0.00) | 1 | (0.04) | - |  |
| *Cardiac disorders* | 1 | (0.19) | 0 | (0.00) | - |  |
| Palpitations | 1 | (0.19) | 0 | (0.00) | - |  |
| *Vascular disorders* | 1 | (0.19) | 2 | (0.08) | - |  |
| Orthostatic hypotension | 0 | (0.00) | 1 | (0.04) | - |  |
| Hypotension | 1 | (0.19) | 1 | (0.04) | - |  |
| *Respiratory, thoracic and mediastinal disorders* | 0 | (0.00) | 1 | (0.04) | - |  |
| Pharyngeal edema | 0 | (0.00) | 1 | (0.04) | - |  |
| *Gastrointestinal disorders* | 3 | (0.56) | 3 | (0.11) | - |  |
| Nausea | 1 | (0.19) | 2 | (0.08) | - |  |
| Gastric ulcer | 1 | (0.19) | 0 | (0.00) | - |  |
| Diarrhea | 0 | (0.00) | 1 | (0.04) | - |  |
| Fecal incontinence | 1 | (0.19) | 0 | (0.00) | - |  |
| *Hepatobiliary disorders* | 0 | (0.00) | 2 | (0.08) | - |  |
| Hepatic function abnormal | 0 | (0.00) | 1 | (0.04) | - |  |
| Liver disorder | 0 | (0.00) | 1 | (0.04) | - |  |
| *Skin and subcutaneous tissue disorders* | 1 | (0.19) | 4 | (0.15) | - |  |
| Photosensitivity reaction | 0 | (0.00) | 1 | (0.04) | - |  |
| Rash | 1 | (0.19) | 2 | (0.08) | - |  |
| Drug eruption | 0 | (0.00) | 1 | (0.04) | - |  |
| *Renal and urinary disorders* | 2 | (0.37) | 8 | (0.30) | - |  |
| Renal impairment | 2 | (0.37) | 5 | (0.19) | - |  |
| Polyuria | 0 | (0.00) | 1 | (0.04) | - |  |
| Frequent urination | 0 | (0.00) | 1 | (0.04) | - |  |
| Nocturia | 0 | (0.00) | 1 | (0.04) | - |  |
| *General disorders and administration site conditions* | 0 | (0.00) | 4 | (0.15) | - |  |
| Malaise | 0 | (0.00) | 1 | (0.04) | - |  |
| Edema | 0 | (0.00) | 1 | (0.04) | - |  |
| Peripheral swelling | 0 | (0.00) | 1 | (0.04) | - |  |
| Drug interaction | 0 | (0.00) | 1 | (0.04) | - |  |
| *Investigations* | 3 | (0.56) | 8 | (0.30) | - |  |
| Blood pressure decreased | 0 | (0.00) | 1 | (0.04) | - |  |
| Blood potassium increased | 3 | (0.56) | 3 | (0.11) | - |  |
| Blood creatinine increased | 0 | (0.00) | 2 | (0.08) | - |  |
| Blood cholesterol increased | 0 | (0.00) | 1 | (0.04) | - |  |
| Blood urea increased | 0 | (0.00) | 1 | (0.04) | - |  |

Calculated with MedDRA/J17.1 System Organ Class and Preferred Terms.

Table S5. Incidence of adverse drug reactions by concomitant antihypertensive medication (direct renin inhibitor) at the time of initiation of eplerenone in safety analysis population (N = 3,166)

| Adverse drug reactions | Concomitant antihypertensive medication | | | | | |
| --- | --- | --- | --- | --- | --- | --- |
|  | With DRI (N (%)) | | WIhtout DRI (N (%)) | | Unknown(N (%)) | |
| Safety analysis population | 13 | | 3153 | | 0 | |
| Patients with adverse drug reactions | 0 | (0.00) | 75 | (2.38) | - |  |
| *Infections and infestations* | 0 | (0.00) | 1 | (0.03) | - |  |
| *Herpes zoster* | 0 | (0.00) | 1 | (0.03) | - |  |
| *Neoplasms (benign, malignant, and unspecified, including cysts and polyps)* | 0 | (0.00) | 1 | (0.03) | - |  |
| Acute myeloid leukemia | 0 | (0.00) | 1 | (0.03) | - |  |
| *Blood and lymphatic system disorders* | 0 | (0.00) | 1 | (0.03) | - |  |
| Lymphadenitis | 0 | (0.00) | 1 | (0.03) | - |  |
| *Metabolism and nutrition disorders* | 0 | (0.00) | 25 | (0.79) | - |  |
| Hyperkalemia | 0 | (0.00) | 20 | (0.63) | - |  |
| Hyperuricemia | 0 | (0.00) | 1 | (0.03) | - |  |
| Decreased appetite | 0 | (0.00) | 1 | (0.03) | - |  |
| Dehydration | 0 | (0.00) | 1 | (0.03) | - |  |
| Hyponatremia | 0 | (0.00) | 2 | (0.06) | - |  |
| *Nervous system disorders* | 0 | (0.00) | 10 | (0.32) | - |  |
| Hypoesthesia | 0 | (0.00) | 1 | (0.03) | - |  |
| Headache | 0 | (0.00) | 1 | (0.03) | - |  |
| Dizziness | 0 | (0.00) | 7 | (0.22) | - |  |
| Convulsion | 0 | (0.00) | 1 | (0.03) | - |  |
| *Ear and labyrinth disorders* | 0 | (0.00) | 1 | (0.03) | - |  |
| Ear congestion | 0 | (0.00) | 1 | (0.03) | - |  |
| *Cardiac disorders* | 0 | (0.00) | 1 | (0.03) | - |  |
| Palpitations | 0 | (0.00) | 1 | (0.03) | - |  |
| *Vascular disorders* | 0 | (0.00) | 3 | (0.10) | - |  |
| Orthostatic hypotension | 0 | (0.00) | 1 | (0.03) | - |  |
| Hypotension | 0 | (0.00) | 2 | (0.06) | - |  |
| *Respiratory, thoracic and mediastinal disorders* | 0 | (0.00) | 1 | (0.03) | - |  |
| Pharyngeal edema | 0 | (0.00) | 1 | (0.03) | - |  |
| *Gastrointestinal disorders* | 0 | (0.00) | 6 | (0.19) | - |  |
| Nausea | 0 | (0.00) | 3 | (0.10) | - |  |
| Gastric ulcer | 0 | (0.00) | 1 | (0.03) | - |  |
| Diarrhea | 0 | (0.00) | 1 | (0.03) | - |  |
| Fecal incontinence | 0 | (0.00) | 1 | (0.03) | - |  |
| *Hepatobiliary disorders* | 0 | (0.00) | 2 | (0.06) | - |  |
| Hepatic function abnormal | 0 | (0.00) | 1 | (0.03) | - |  |
| Liver disorder | 0 | (0.00) | 1 | (0.03) | - |  |
| *Skin and subcutaneous tissue disorders* | 0 | (0.00) | 5 | (0.16) | - |  |
| Photosensitivity reaction | 0 | (0.00) | 1 | (0.03) | - |  |
| Rash | 0 | (0.00) | 3 | (0.10) | - |  |
| Drug eruption | 0 | (0.00) | 1 | (0.03) | - |  |
| *Renal and urinary disorders* | 0 | (0.00) | 10 | (0.32) | - |  |
| Renal impairment | 0 | (0.00) | 7 | (0.22) | - |  |
| Polyuria | 0 | (0.00) | 1 | (0.03) | - |  |
| Frequent urination | 0 | (0.00) | 1 | (0.03) | - |  |
| Nocturia | 0 | (0.00) | 1 | (0.03) | - |  |
| *General disorders and administration site conditions* | 0 | (0.00) | 4 | (0.13) | - |  |
| Malaise | 0 | (0.00) | 1 | (0.03) | - |  |
| Edema | 0 | (0.00) | 1 | (0.03) | - |  |
| Peripheral swelling | 0 | (0.00) | 1 | (0.03) | - |  |
| Drug interaction | 0 | (0.00) | 1 | (0.03) | - |  |
| *Investigations* | 0 | (0.00) | 11 | (0.35) | - |  |
| Blood pressure decreased | 0 | (0.00) | 1 | (0.03) | - |  |
| Blood potassium increased | 0 | (0.00) | 6 | (0.19) | - |  |
| Blood creatinine increased | 0 | (0.00) | 2 | (0.06) | - |  |
| Blood cholesterol increased | 0 | (0.00) | 1 | (0.03) | - |  |
| Blood urea increased | 0 | (0.00) | 1 | (0.03) | - |  |

Calculated with MedDRA/J17.1 System Organ Class and Preferred Terms. DRI: direct renin inhibitor

Table S6. Incidence of adverse drug reactions by concomitant antihypertensive medication (angiotensin converting enzyme inhibitor) at the time of initiation of eplerenone in safety analysis population (N = 3,166)

| Adverse drug reactions | Concomitant antihypertensive medication | | | | | |
| --- | --- | --- | --- | --- | --- | --- |
|  | With ACEI (N (%)) | | Without ACEI (N (%)) | | Unknown(N (%)) | |
| Safety analysis population | 268 | | 2898 | | 0 | |
| Patients with adverse drug reactions | 7 | (2.61) | 68 | (2.35) | - |  |
| *Infections and infestations* | 0 | (0.00) | 1 | (0.03) | - |  |
| *Herpes zoster* | 0 | (0.00) | 1 | (0.03) | - |  |
| *Neoplasms (benign, malignant, and unspecified, including cysts and polyps)* | 0 | (0.00) | 1 | (0.03) | - |  |
| Acute myeloid leukemia | 0 | (0.00) | 1 | (0.03) | - |  |
| *Blood and lymphatic system disorders* | 0 | (0.00) | 1 | (0.03) | - |  |
| Lymphadenitis | 0 | (0.00) | 1 | (0.03) | - |  |
| *Metabolism and nutrition disorders* | 3 | (1.12) | 22 | (0.76) | - |  |
| Hyperkalemia | 3 | (1.12) | 17 | (0.59) | - |  |
| Hyperuricemia | 0 | (0.00) | 1 | (0.03) | - |  |
| Decreased appetite | 0 | (0.00) | 1 | (0.03) | - |  |
| Dehydration | 0 | (0.00) | 1 | (0.03) | - |  |
| Hyponatremia | 0 | (0.00) | 2 | (0.07) | - |  |
| *Nervous system disorders* | 0 | (0.00) | 10 | (0.35) | - |  |
| Hypoesthesia | 0 | (0.00) | 1 | (0.03) | - |  |
| Headache | 0 | (0.00) | 1 | (0.03) | - |  |
| Dizziness | 0 | (0.00) | 7 | (0.24) | - |  |
| Convulsion | 0 | (0.00) | 1 | (0.03) | - |  |
| *Ear and labyrinth disorders* | 0 | (0.00) | 1 | (0.03) | - |  |
| Ear congestion | 0 | (0.00) | 1 | (0.03) | - |  |
| *Cardiac disorders* | 0 | (0.00) | 1 | (0.03) | - |  |
| Palpitations | 0 | (0.00) | 1 | (0.03) | - |  |
| *Vascular disorders* | 0 | (0.00) | 3 | (0.10) | - |  |
| Orthostatic hypotension | 0 | (0.00) | 1 | (0.03) | - |  |
| Hypotension | 0 | (0.00) | 2 | (0.07) | - |  |
| *Respiratory, thoracic and mediastinal disorders* | 0 | (0.00) | 1 | (0.03) | - |  |
| Pharyngeal edema | 0 | (0.00) | 1 | (0.03) | - |  |
| *Gastrointestinal disorders* | 3 | (1.12) | 3 | (0.10) | - |  |
| Nausea | 2 | (0.75) | 1 | (0.03) | - |  |
| Gastric ulcer | 1 | (0.37) | 0 | (0.00) | - |  |
| Diarrhea | 0 | (0.00) | 1 | (0.03) | - |  |
| Fecal incontinence | 0 | (0.00) | 1 | (0.03) | - |  |
| *Hepatobiliary disorders* | 0 | (0.00) | 2 | (0.07) | - |  |
| Hepatic function abnormal | 0 | (0.00) | 1 | (0.03) | - |  |
| Liver disorder | 0 | (0.00) | 1 | (0.03) | - |  |
| *Skin and subcutaneous tissue disorders* | 0 | (0.00) | 5 | (0.17) | - |  |
| Photosensitivity reaction | 0 | (0.00) | 1 | (0.03) | - |  |
| Rash | 0 | (0.00) | 3 | (0.10) | - |  |
| Drug eruption | 0 | (0.00) | 1 | (0.03) | - |  |
| *Renal and urinary disorders* | 0 | (0.00) | 10 | (0.35) | - |  |
| Renal impairment | 0 | (0.00) | 7 | (0.24) | - |  |
| Polyuria | 0 | (0.00) | 1 | (0.03) | - |  |
| Frequent urination | 0 | (0.00) | 1 | (0.03) | - |  |
| Nocturia | 0 | (0.00) | 1 | (0.03) | - |  |
| *General disorders and administration site conditions* | 1 | (0.37) | 3 | (0.10) | - |  |
| Malaise | 0 | (0.00) | 1 | (0.03) | - |  |
| Edema | 0 | (0.00) | 1 | (0.03) | - |  |
| Peripheral swelling | 0 | (0.00) | 1 | (0.03) | - |  |
| Drug interaction | 1 | (0.37) | 0 | (0.00) | - |  |
| *Investigations* | 1 | (0.37) | 10 | (0.35) | - |  |
| Blood pressure decreased | 0 | (0.00) | 1 | (0.03) | - |  |
| Blood potassium increased | 1 | (0.37) | 5 | (0.17) | - |  |
| Blood creatinine increased | 0 | (0.00) | 2 | (0.07) | - |  |
| Blood cholesterol increased | 0 | (0.00) | 1 | (0.03) | - |  |
| Blood urea increased | 0 | (0.00) | 1 | (0.03) | - |  |

Calculated with MedDRA/J17.1　System Organ Class and Preferred Terms. ACEI: angiotensin-converting enzyme inhibitor

Table S7. Incidence of adverse drug reactions by concomitant antihypertensive medication (Angiotensin II Receptor Blocker) at the time of initiation of eplerenone in safety analysis population (N = 3,166)

| Adverse drug reactions | Concomitant antihypertensive medication | | | | | |
| --- | --- | --- | --- | --- | --- | --- |
|  | With ARB (N (%)) | | Without ARB (N (%)) | | Unknown(N (%)) | |
| Safety analysis population | 1476 | | 1690 | | 0 | |
| Patients with adverse drug reactions | 42 | (2.85) | 33 | (1.95) | - |  |
| *Infections and infestations* | 0 | (0.00) | 1 | (0.06) | - |  |
| *Herpes zoster* | 0 | (0.00) | 1 | (0.06) | - |  |
| *Neoplasms (benign, malignant, and unspecified, including cysts and polyps)* | 1 | (0.07) | 0 | (0.00) | - |  |
| Acute myeloid leukemia | 1 | (0.07) | 0 | (0.00) | - |  |
| *Blood and lymphatic system disorders* | 0 | (0.00) | 1 | (0.06) | - |  |
| Lymphadenitis | 0 | (0.00) | 1 | (0.06) | - |  |
| *Metabolism and nutrition disorders* | 13 | (0.88) | 12 | (0.71) | - |  |
| Hyperkalemia | 11 | (0.75) | 9 | (0.53) | - |  |
| Hyperuricemia | 1 | (0.07) | 0 | (0.00) | - |  |
| Decreased appetite | 0 | (0.00) | 1 | (0.06) | - |  |
| Dehydration | 1 | (0.07) | 0 | (0.00) | - |  |
| Hyponatremia | 0 | (0.00) | 2 | (0.12) | - |  |
| *Nervous system disorders* | 4 | (0.27) | 6 | (0.36) | - |  |
| Hypoesthesia | 1 | (0.07) | 0 | (0.00) | - |  |
| Headache | 0 | (0.00) | 1 | (0.06) | - |  |
| Dizziness | 2 | (0.14) | 5 | (0.30) | - |  |
| Convulsion | 1 | (0.07) | 0 | (0.00) | - |  |
| *Ear and labyrinth disorders* | 1 | (0.07) | 0 | (0.00) | - |  |
| Ear congestion | 1 | (0.07) | 0 | (0.00) | - |  |
| *Cardiac disorders* | 0 | (0.00) | 1 | (0.06) | - |  |
| Palpitations | 0 | (0.00) | 1 | (0.06) | - |  |
| *Vascular disorders* | 2 | (0.14) | 1 | (0.06) | - |  |
| Orthostatic hypotension | 0 | (0.00) | 1 | (0.06) | - |  |
| Hypotension | 2 | (0.14) | 0 | (0.00) | - |  |
| *Respiratory, thoracic and mediastinal disorders* | 0 | (0.00) | 1 | (0.06) | - |  |
| Pharyngeal edema | 0 | (0.00) | 1 | (0.06) | - |  |
| *Gastrointestinal disorders* | 2 | (0.14) | 4 | (0.24) | - |  |
| Nausea | 2 | (0.14) | 1 | (0.06) | - |  |
| Gastric ulcer | 0 | (0.00) | 1 | (0.06) | - |  |
| Diarrhea | 0 | (0.00) | 1 | (0.06) | - |  |
| Fecal incontinence | 0 | (0.00) | 1 | (0.06) | - |  |
| *Hepatobiliary disorders* | 1 | (0.07) | 1 | (0.06) | - |  |
| Hepatic function abnormal | 0 | (0.00) | 1 | (0.06) | - |  |
| Liver disorder | 1 | (0.07) | 0 | (0.00) | - |  |
| *Skin and subcutaneous tissue disorders* | 3 | (0.20) | 2 | (0.12) | - |  |
| Photosensitivity reaction | 1 | (0.07) | 0 | (0.00) | - |  |
| Rash | 2 | (0.14) | 1 | (0.06) | - |  |
| Drug eruption | 0 | (0.00) | 1 | (0.06) | - |  |
| *Renal and urinary disorders* | 8 | (0.54) | 2 | (0.12) | - |  |
| Renal impairment | 6 | (0.41) | 1 | (0.06) | - |  |
| Polyuria | 0 | (0.00) | 1 | (0.06) | - |  |
| Frequent urination | 1 | (0.07) | 0 | (0.00) | - |  |
| Nocturia | 1 | (0.07) | 0 | (0.00) | - |  |
| *General disorders and administration site conditions* | 3 | (0.20) | 1 | (0.06) | - |  |
| Malaise | 1 | (0.07) | 0 | (0.00) | - |  |
| Edema | 0 | (0.00) | 1 | (0.06) | - |  |
| Peripheral swelling | 1 | (0.07) | 0 | (0.00) | - |  |
| Drug interaction | 1 | (0.07) | 0 | (0.00) | - |  |
| *Investigations* | 7 | (0.47) | 4 | (0.24) | - |  |
| Blood pressure decreased | 1 | (0.07) | 0 | (0.00) | - |  |
| Blood potassium increased | 4 | (0.27) | 2 | (0.12) | - |  |
| Blood creatinine increased | 1 | (0.07) | 1 | (0.06) | - |  |
| Blood cholesterol increased | 0 | (0.00) | 1 | (0.06) | - |  |
| Blood urea increased | 1 | (0.07) | 0 | (0.00) | - |  |

Calculated with MedDRA/J17.1　System Organ Class and Preferred Terms. ARB: angiotensin II receptor blocker

Table S8. Incidence of adverse drug reactions by concomitant antihypertensive medication (thiazide diuretics) at the time of initiation of eplerenone in safety analysis population (N = 3,166)

| Adverse drug reactions | Concomitant antihypertensive medication | | | | | |
| --- | --- | --- | --- | --- | --- | --- |
|  | With thiazide diuretics (N (%)) | | Without thiazide diuretics (N (%)) | | Unknown(N (%)) | |
| Safety analysis population | 305 | | 2861 | | 0 | |
| Patients with adverse drug reactions | 4 | (1.31) | 71 | (2.48) | - |  |
| *Infections and infestations* | 0 | (0.00) | 1 | (0.03) | - |  |
| *Herpes zoster* | 0 | (0.00) | 1 | (0.03) | - |  |
| *Neoplasms (benign, malignant, and unspecified, including cysts and polyps)* | 0 | (0.00) | 1 | (0.03) | - |  |
| Acute myeloid leukemia | 0 | (0.00) | 1 | (0.03) | - |  |
| *Blood and lymphatic system disorders* | 0 | (0.00) | 1 | (0.03) | - |  |
| Lymphadenitis | 0 | (0.00) | 1 | (0.03) | - |  |
| *Metabolism and nutrition disorders* | 0 | (0.00) | 25 | (0.87) | - |  |
| Hyperkalemia | 0 | (0.00) | 20 | (0.70) | - |  |
| Hyperuricemia | 0 | (0.00) | 1 | (0.03) | - |  |
| Decreased appetite | 0 | (0.00) | 1 | (0.03) | - |  |
| Dehydration | 0 | (0.00) | 1 | (0.03) | - |  |
| Hyponatremia | 0 | (0.00) | 2 | (0.07) | - |  |
| *Nervous system disorders* | 0 | (0.00) | 10 | (0.35) | - |  |
| Hypoesthesia | 0 | (0.00) | 1 | (0.03) | - |  |
| Headache | 0 | (0.00) | 1 | (0.03) | - |  |
| Dizziness | 0 | (0.00) | 7 | (0.24) | - |  |
| Convulsion | 0 | (0.00) | 1 | (0.03) | - |  |
| *Ear and labyrinth disorders* | 1 | (0.33) | 0 | (0.00) | - |  |
| Ear congestion | 1 | (0.33) | 0 | (0.00) | - |  |
| *Cardiac disorders* | 0 | (0.00) | 1 | (0.03) | - |  |
| Palpitations | 0 | (0.00) | 1 | (0.03) | - |  |
| *Vascular disorders* | 1 | (0.33) | 2 | (0.07) | - |  |
| Orthostatic hypotension | 0 | (0.00) | 1 | (0.03) | - |  |
| Hypotension | 1 | (0.33) | 1 | (0.03) | - |  |
| *Respiratory, thoracic and mediastinal disorders* | 0 | (0.00) | 1 | (0.03) | - |  |
| Pharyngeal edema | 0 | (0.00) | 1 | (0.03) | - |  |
| *Gastrointestinal disorders* | 0 | (0.00) | 6 | (0.21) | - |  |
| Nausea | 0 | (0.00) | 3 | (0.10) | - |  |
| Gastric ulcer | 0 | (0.00) | 1 | (0.03) | - |  |
| Diarrhea | 0 | (0.00) | 1 | (0.03) | - |  |
| Fecal incontinence | 0 | (0.00) | 1 | (0.03) | - |  |
| *Hepatobiliary disorders* | 0 | (0.00) | 2 | (0.07) | - |  |
| Hepatic function abnormal | 0 | (0.00) | 1 | (0.03) | - |  |
| Liver disorder | 0 | (0.00) | 1 | (0.03) | - |  |
| *Skin and subcutaneous tissue disorders* | 0 | (0.00) | 5 | (0.17) | - |  |
| Photosensitivity reaction | 0 | (0.00) | 1 | (0.03) | - |  |
| Rash | 0 | (0.00) | 3 | (0.10) | - |  |
| Drug eruption | 0 | (0.00) | 1 | (0.03) | - |  |
| *Renal and urinary disorders* | 1 | (0.33) | 9 | (0.31) | - |  |
| Renal impairment | 1 | (0.33) | 6 | (0.21) | - |  |
| Polyuria | 0 | (0.00) | 1 | (0.03) | - |  |
| Frequent urination | 0 | (0.00) | 1 | (0.03) | - |  |
| Nocturia | 0 | (0.00) | 1 | (0.03) | - |  |
| *General disorders and administration site conditions* | 0 | (0.00) | 4 | (0.14) | - |  |
| Malaise | 0 | (0.00) | 1 | (0.03) | - |  |
| Edema | 0 | (0.00) | 1 | (0.03) | - |  |
| Peripheral swelling | 0 | (0.00) | 1 | (0.03) | - |  |
| Drug interaction | 0 | (0.00) | 1 | (0.03) | - |  |
| *Investigations* | 1 | (0.33) | 10 | (0.35) | - |  |
| Blood pressure decreased | 0 | (0.00) | 1 | (0.03) | - |  |
| Blood potassium increased | 0 | (0.00) | 6 | (0.21) | - |  |
| Blood creatinine increased | 1 | (0.33) | 1 | (0.03) | - |  |
| Blood cholesterol increased | 0 | (0.00) | 1 | (0.03) | - |  |
| Blood urea increased | 0 | (0.00) | 1 | (0.03) | - |  |

Calculated with MedDRA/J17.1　System Organ Class and Preferred Terms.

Table S9. Incidence of adverse drug reactions by concomitant antihypertensive medication (loop diuretics) at the time of initiation of eplerenone in safety analysis population (N = 3,166)

| Adverse drug reactions | Concomitant antihypertensive medication | | | | | |
| --- | --- | --- | --- | --- | --- | --- |
|  | With loop diuretics (N (%)) | | Without loop diuretics (N (%)) | | Unknown (N (%)) | |
| Safety analysis population | 355 | | 2811 | | 0 | |
| Patients with adverse drug reactions | 11 | (3.10) | 64 | (2.28) | - |  |
| *Infections and infestations* | 0 | (0.00) | 1 | (0.04) | - |  |
| *Herpes zoster* | 0 | (0.00) | 1 | (0.04) | - |  |
| *Neoplasms (benign, malignant, and unspecified, including cysts and polyps)* | 0 | (0.00) | 1 | (0.04) | - |  |
| Acute myeloid leukemia | 0 | (0.00) | 1 | (0.04) | - |  |
| *Blood and lymphatic system disorders* | 0 | (0.00) | 1 | (0.04) | - |  |
| Lymphadenitis | 0 | (0.00) | 1 | (0.04) | - |  |
| *Metabolism and nutrition disorders* | 3 | (0.85) | 22 | (0.78) | - |  |
| Hyperkalemia | 3 | (0.85) | 17 | (0.60) | - |  |
| Hyperuricemia | 0 | (0.00) | 1 | (0.04) | - |  |
| Decreased appetite | 0 | (0.00) | 1 | (0.04) | - |  |
| Dehydration | 0 | (0.00) | 1 | (0.04) | - |  |
| Hyponatremia | 0 | (0.00) | 2 | (0.07) | - |  |
| *Nervous system disorders* | 0 | (0.00) | 10 | (0.36) | - |  |
| Hypoesthesia | 0 | (0.00) | 1 | (0.04) | - |  |
| Headache | 0 | (0.00) | 1 | (0.04) | - |  |
| Dizziness | 0 | (0.00) | 7 | (0.25) | - |  |
| Convulsion | 0 | (0.00) | 1 | (0.04) | - |  |
| *Ear and labyrinth disorders* | 0 | (0.00) | 1 | (0.04) | - |  |
| Ear congestion | 0 | (0.00) | 1 | (0.04) | - |  |
| *Cardiac disorders* | 0 | (0.00) | 1 | (0.04) | - |  |
| Palpitations | 0 | (0.00) | 1 | (0.04) | - |  |
| *Vascular disorders* | 1 | (0.28) | 2 | (0.07) | - |  |
| Orthostatic hypotension | 0 | (0.00) | 1 | (0.04) | - |  |
| Hypotension | 1 | (0.28) | 1 | (0.04) | - |  |
| *Respiratory, thoracic and mediastinal disorders* | 0 | (0.00) | 1 | (0.04) | - |  |
| Pharyngeal edema | 0 | (0.00) | 1 | (0.04) | - |  |
| *Gastrointestinal disorders* | 1 | (0.28) | 5 | (0.18) | - |  |
| Nausea | 0 | (0.00) | 3 | (0.11) | - |  |
| Gastric ulcer | 1 | (0.28) | 0 | (0.00) | - |  |
| Diarrhea | 0 | (0.00) | 1 | (0.04) | - |  |
| Fecal incontinence | 0 | (0.00) | 1 | (0.04) | - |  |
| *Hepatobiliary disorders* | 0 | (0.00) | 2 | (0.07) | - |  |
| Hepatic function abnormal | 0 | (0.00) | 1 | (0.04) | - |  |
| Liver disorder | 0 | (0.00) | 1 | (0.04) | - |  |
| *Skin and subcutaneous tissue disorders* | 1 | (0.28) | 4 | (0.14) | - |  |
| Photosensitivity reaction | 0 | (0.00) | 1 | (0.04) | - |  |
| Rash | 1 | (0.28) | 2 | (0.07) | - |  |
| Drug eruption | 0 | (0.00) | 1 | (0.04) | - |  |
| *Renal and urinary disorders* | 3 | (0.85) | 7 | (0.25) | - |  |
| Renal impairment | 3 | (0.85) | 4 | (0.14) | - |  |
| Polyuria | 0 | (0.00) | 1 | (0.04) | - |  |
| Frequent urination | 0 | (0.00) | 1 | (0.04) | - |  |
| Nocturia | 0 | (0.00) | 1 | (0.04) | - |  |
| *General disorders and administration site conditions* | 0 | (0.00) | 4 | (0.14) | - |  |
| Malaise | 0 | (0.00) | 1 | (0.04) | - |  |
| Edema | 0 | (0.00) | 1 | (0.04) | - |  |
| Peripheral swelling | 0 | (0.00) | 1 | (0.04) | - |  |
| Drug interaction | 0 | (0.00) | 1 | (0.04) | - |  |
| *Investigations* | 2 | (0.56) | 9 | (0.32) | - |  |
| Blood pressure decreased | 0 | (0.00) | 1 | (0.04) | - |  |
| Blood potassium increased | 2 | (0.56) | 4 | (0.14) | - |  |
| Blood creatinine increased | 0 | (0.00) | 2 | (0.07) | - |  |
| Blood cholesterol increased | 0 | (0.00) | 1 | (0.04) | - |  |
| Blood urea increased | 0 | (0.00) | 1 | (0.04) | - |  |

Calculated with MedDRA/J17.1　System Organ Class and Preferred Terms.
